# Gut Microbiota and Other Factors Associated With Increased Regulatory T Cells in Hiv-exposed Uninfected Infants

**DOI:** 10.21203/rs.3.rs-3909424/v1

**Published:** 2024-02-02

**Authors:** Michael Johnson, Sarah K. Lazarus, Ashlynn E. Bennett, Adriana Tovar-Salazar, Charles E. Robertson, Jennifer M. Kofonow, Shaobing Li, Bruce McCollister, Marta C. Nunes, Shabir A. Madhi, Daniel N. Frank, Adriana Weinberg

## Abstract

HIV-exposed uninfected infants (HEU) have higher infectious morbidity than HIV-unexposed infants (HUU). HEU have multiple immune defects of unknown origin. We hypothesized that HEU have higher regulatory T cells (Treg) than HUU, which may dampen their immune defenses against pathogens. We compared 25 Treg subsets between HEU and HUU and sought the factors that may affect Treg frequencies. At birth, 3 Treg subsets, including CD4 + FOXP3 + and CD4 + FOXP3 + CD25+, had higher frequencies in 123 HEU than 117 HUU and 3 subsets were higher in HUU. At 28 and 62 weeks of life, 5 Treg subsets were higher in HEU, and none were higher in HUU. The frequencies of the discrepant Treg subsets correlated at birth with differential abundances of bacterial taxas in maternal gut microbiome and at subsequent visits in infant gut microbiomes. In vitro, bacterial taxa most abundant in HEU expanded Treg subsets with higher frequencies in HEU, recapitulating the in vivo observations. Other factors that correlated with increased Treg were low maternal CD4 + T cells in HEU at birth and male sex in HUU at 28 weeks. We conclude that maternal and infant gut dysbiosis are central to the Treg increase in HEU and may be targeted by mitigating interventions.

## Introduction

Due to extraordinary advances in the prevention of HIV vertical transmission, 2 million HIV-exposed uninfected infants (HEU) are born every year. However, compared with HIV-unexposed infants (HUU), HEU have a higher incidence of hospitalization and death due to severe infections during the first 1–2 years of life [1–3]. The introduction of universal 3-drug antiretroviral therapy (ART) during pregnancy in 2012 moderately improved clinical and infectious outcomes of HEU, but growth deficits and increased hospitalizations in early childhood continued to be reported more than five years after the 3-drug ART implementation[4–8]. In the US and other countries of the Northern hemisphere, hospitalizations, but not mortality, are increased in HEU compared to HUU [9, 10] probably due to advanced health care capabilities.

The infections with high morbidity and mortality in HEU are caused by common childhood pathogens, including respiratory viruses and *S. pneumoniae* [11–16]. It was proposed that insufficient passive immune protection due to low transplacental transport of maternal antibodies in pregnant people with HIV accounted for the high severity of infections in HEU [13, 17]. However, we showed that antibody concentrations at birth against a variety of respiratory pathogens, including viruses and *S. pneumoniae*, were similar in HEU who developed medically attended lower respiratory tract infections in the first 6 months of life and those who did not[18]. Moreover, maternal antibodies decay during the first 2–3 months of life below protective levels [19, 20] and, therefore, insufficient maternal antibodies could not explain higher susceptibility to infection in HEU beyond the first 3 months of life.

Multiple immunologic abnormalities identified in HEU may contribute to their increased rate of severe infection, hospitalization, and death. Due to the broad spectrum of regulatory T cell (Treg) activity against multiple immune cell subsets, this observation provides a potential unifying mechanism for the increased susceptibility to severe infections in HEU. People with HIV also have multiple immunologic abnormalities, including excessive frequencies of Treg, which have been associated with accelerated disease progression and high susceptibility to severe infections [21–24]. In the general population, Treg have been associated with decreased immune protection against tumors and viral infections [25–27]. We hypothesized that enhanced Treg frequencies in HEU may increase their susceptibility to infections in early childhood.

The Treg hallmark is the transcription factor FOXP3, which inhibits *Ifng* and *Il2* gene transcriptions and thereby prevents conventional T cell differentiation [28]. However, multiple other Treg subsets have been described based on functional and phenotypic markers, including some that we have reported to differ between HEU and HUU at birth [29]. The goal of this study was to undertake a comprehensive analysis of the relative abundance of Treg subsets in HEU and HUU during the first year of life and identify factors associated with differences between the two infant groups, including maternal HIV disease characteristics, infant demographics, epigenomics, and gut microbiome composition.

## Results

<u>Characteristics of the study population</u>

This study enrolled 240 mother-infant pairs including 123 mothers with HIV and 117 without HIV between June and December 2017. Notable differences between mothers in the two groups were higher chronological age and parity, and lower body mass index (BMI) in mothers with HIV (**Table 1**).

There were no differences in alcohol or tobacco use or level of education between the two groups. Mothers with HIV received ART during pregnancy, had a median of 347 CD4+ T cells/µl of blood, and <50 HIV RNA copies/ml of plasma.

At birth, HEU and HUU had similar gestational ages by study design with an average of 39 weeks. The sex distribution was also similar (**Table 1**). HEU had significantly lower birth weight with a mean of 3070g compared with 3250g in HUU. None of the infants were small or large for gestational age. Seven HEU and one HUU were delivered by emergency C-section for obstetrical indications identified after initiation of labor.

Infant diet and antibiotic usage were recorded at each visit due to their relevance to the gut microbiome composition (**Table S1**). At 6 weeks of life, >80% of infants were exclusively breastfed in each group and 13% of HEU and 5% of HUU, respectively, were exclusively formula-fed. At 28 weeks, 61% of HEU and 50% of HUU were still exclusively breastfed, whereas exclusive formula-feeding was reported in 22% of HEU and 11% of HUU. At 62 weeks, all infants received solid foods. Cotrimoxazole was used in 5%, 90% and 87% of the HEU at 6, 28 and 62 weeks of life (**Table S1**).

**Table 1 - Participant characteristics at Delivery#**

| Mothers | Mothers with HIV | Mothers without HIV | p-value |
|---|---|---|---|
| | (N=123) | (N=117) | |
| **Age (years)** | | | |
| Median [Q1, Q3] | 30.0 [26.0, 34.5] | 25.0 [22.0, 30.0] | < 0.01 |
| **Previous Pregnancies** | | | |
| Median [Q1, Q3] | 2.00 [1.00, 3.00] | 1.00 [0, 2.00] | < 0.01 |
| Missing | 2 (1.6%) | 5 (4.3%) | |
| **BMI at 62 Weeks** | | | |
| Median [Q1, Q3] | 23.9 [20.5, 28.1] | 27.1 [21.7, 31.1] | 0.02 |
| Missing | 51 (41.5%) | 59 (50.4%) | |
| **Smoking During Pregnancy** | | | |
| No | 114 (92.7%) | 113 (96.6%) | 0.18 |
| **Alcohol During Pregnancy** | | | |
| No | 112 (91.1%) | 109 (93.2%) | 0.55 |
| **CD4+ Cells/μl** | | | |
| Median [Q1, Q3] | 347 [227, 499] | NA | |
| Missing | 7 (5.7%) | NA | |
| **Log HIV RNA copies/ml** | | | |
| Median [Q1, Q3] | 1.00 [0, 2.06]* | NA | |
| Missing | 10 (8.1%) | NA | |
| **Compliant with ART** | | | |
| Yes | 122 (99.2%) | NA | |

| Infants | HEU | HUU | |
|---|---|---|---|
| | (N=123) | (N=117) | |
| **Sex** | | | |
| Female | 65 (52.8%) | 56 (47.9%) | 0.44 |
| **Mode of Delivery** | | | |
| Vaginal | 116 (94.3%) | 116 (99.1%) | 0.07 |

| Gestational Age (weeks) | | | |
|---|---|---|---|
| Mean (SD) | 39.1 (2.22) | 39.3 (1.52) | 0.48 |
| Missing | 4 (3.3%) | 1 (0.9%) | |
| **Birth Weight (g)** | | | |
| Mean (SD) | 3070 (423) | 3250 (437) | < 0.01 |

##Maternal BMI was measured at 62 weeks postpartum.

*Target not detected was assigned a numeric of 0 and <20 a value of 10 copies/ml.

**Abbreviations**: ART = antiretroviral treatment; BMI = Body mass index; HIV = Human immunodeficiency virus; HEU = HIV-exposed uninfected: HUU = HIV-unexposed uninfected.

Treg subset distribution in HEU and HUU

CD4+ and CD8+ Treg subsets were identified by the expression of FOXP3 and/or CD25, CD39, CTLA4, GITR, granzyme B (GranzB), IL10, IL35, LAG3, PD1, TGFb, TIM3, TIGIT, and/or TNFR2 using two 10-color flow cytometry panels referred to here as panels A and B (gating strategy shown in **Figure S1**). Treg subsets with frequencies <0.001 at all time points were excluded from the analyses through an a priori decision based on the analytical sensitivity of the flow cytometry method. The comparison of Treg subsets in cord blood between the two groups (**Figure 1; Table S2**) showed significantly higher proportions of CD4+FOXP3+, CD4+FOXP3+CD25+, and CD4+GITR+ Treg in HEU than HUU and higher proportions of CD4+FOXP3+GranzB+, CD4+TGFb+, and CD8+TGFb+ in HUU than HEU [false discovery rate (FDR)-adjusted p <0.1].

There were no significant differences at 6 weeks of life. At 28 and 62 weeks, Treg subsets that significantly differed between the two groups were invariably higher in HEU than HUU and included CD4+GITR+, CD4+IL35+, CD4+TGFb+, CD8+IL35+, and CD8+TGFb+.

Six Treg subsets had significantly different trajectories over time in HEU compared to HUU, including CD4+FOXP3+, CD4+FOXP3+CD25+, CD8+CD25+, CD8+IL10+, CD4+TGFb+, and CD8+TGFb+ (not depicted). Changes over time were consistent with the proportions of the Treg subsets at each time point presented in **Figure 1**.

**Figure 1. Comparison of Treg subset frequencies in HEU and HUU.** Data were derived from a longitudinal cohort of 123 HEU and 117 HUU. Treg subsets listed on the y ordinate were compared between HEU and HUU using Wilcoxon rank-sum test. The dots represent differences in each Treg subset at the time points indicated on the graph (top). N (bottom) indicates the number of HEU in red font and HUU in blue font that contributed data at each time point. Red dots indicate significant differences with FDR-adjusted p<0.1. The size of each dot is inversely proportional to the unadjusted p value. The distance between each dot and 0 is proportional to the size of the estimated difference. Please see **Figure S2** for the gating strategy and **Table S2** for a listing of medians and p values.

Relationship between Treg subsets

To understand if the coordination among Treg subsets differed in HEU from HUU, we performed correlation analyses at each time point between the subsets measured in each flow cytometry panel. Results were visualized as networks displaying positive or negative Spearman correlations among the Treg subsets (**Figure 2, Figure S2**). The analysis of the subsets in flow panel A showed that FOXP3+ and CD25+ Treg subsets were generally positively associated at all time points and in both groups (CD25/FOXP3 cluster). Another relatively stable cluster across groups and time points included Lag3+ and CTLA4+ Treg subsets (Lag3/CTLA4 cluster). The CD25/FOXP3 and Lag3/CTLA4 clusters showed negative correlations with each other at birth and positive correlations at subsequent time points in both groups. CD4+IL10+ and CD8+IL10+ Treg subsets positively correlated with each other (IL10 cluster) at all time points in both groups, as well as CD4+TGFb+ and CD8+TGFb+ (TGFb cluster). In HEU, the IL10 and TGFb clusters positively correlated at all time points. In HUU, the correlations between the IL10 and TGFb clusters alternated between positive and negative at different time points. Overall, the IL10 and TGFb clusters negatively correlated with CD25/FOXP3 clusters in both groups. Thus, the correlation analyses revealed mostly similar relationships between Treg subsets in HEU and HUU. Correlation analyses among Treg subsets in Panel B (**Figure S2**) did not reveal stable patterns or differences between groups over time.

**Figure 2. Relationship between Treg subsets in HEU and HUU.** Data were derived from 29 to 100 participants per group. Red cords indicate positive correlations and blue negative correlations. The color in tensity is proportional to the absolute magnitude of the Spearman correlation coefficient. Treg subsets belonging to the same cluster are identified by the color of their names. Most clusters are conserved across time points in HEU and HUU. Please **Figure S3** for correlations of additional Treg subsets.

Effect of maternal HIV disease characteristics on the distribution of Treg in HEU

We investigated the relationship of maternal CD4+ cell numbers and plasma HIV RNA copies/ml at delivery with the frequency of Treg subsets in HEU. The Spearman correlation analysis of CD4+ cell numbers with all Treg subsets at all visits revealed significant correlations only at birth and only for three subsets: CD4+TGFb+, CD8+TGFb+ and CD8+CTLA4+ (**Figure 3**). The frequencies of the three Treg subsets increased with lower maternal CD4+ cell numbers with rho of -0.27 to -0.35 and adjusted p values of 0.03 to 0.08. There were no differences in the Treg subset frequencies between HEU born to mothers with HIV plasma RNA <50 copies/ml or ≥50 copies/ml (not depicted).

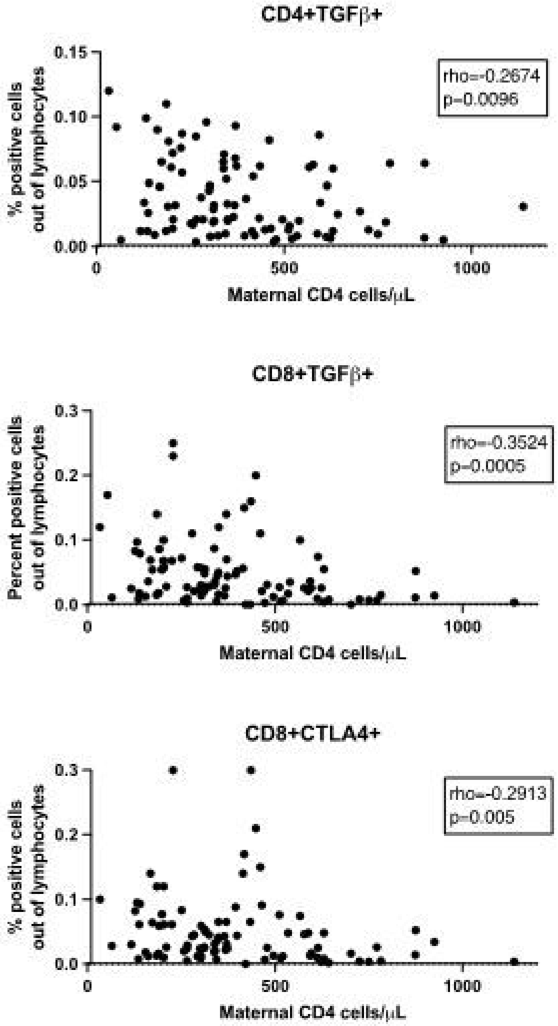

**Figure 3. Effect of maternal CD4+ cell numbers on HEU Treg subsets**. Data were derived from 99 HEU. Graphs show the correlations between maternal CD4+ cell numbers at delivery and frequencies of the Treg subsets denoted in the title of each graph. The graphs display coefficient of correlations and raw p values calculated by Spearman's test. FDR p values were ≤0.8.

Differential DNA methylation of CD4+ T cells in HEU and HUU at birth did not explain Treg variances

We hypothesized that differential Treg distributions between HEU and HUU starting at birth might reflect variability in patterns of DNA methylation acquired in utero. This hypothesis seemed particularly appropriate to explain the excess expression of FOXP3 in HEU CD4+ T cells, which has been associated with hypomethylation of several DNA loci[28]. However, the analysis of differentially methylated regions (DMR) in CD4+ T cells from cord blood of 40 HEU and 40 HUU found significant differences in a single gene, *thioredoxin-interacting protein* (*TXNIP*), which was hypomethylated in HEU compared HUU (**Table S3**). Pathway analyses using GO and KEGG databases did not reveal any significant differences. Roles of *TXNIP* products have been described in hematopoietic cell differentiation, proliferation, apoptosis, and NK cell function[30, 31], but a direct contribution to the differentiation of Treg has not been identified to date.

Effect of infant demographic characteristics on the frequencies of Treg

We investigated the relationship of birth weight and sex with the Treg distribution. We did not find any relationship between birth weight and Treg frequencies in HEU or HUU (not depicted). We found a significant effect of sex only in HUU and only at 28 weeks of life with males showing higher proportions of CD4+FOXP3+GranzB+ and CD8+FOXP3+ Treg (**Figure 4**). Because all infants were born at term by study design, we could not study the effect of gestational age.

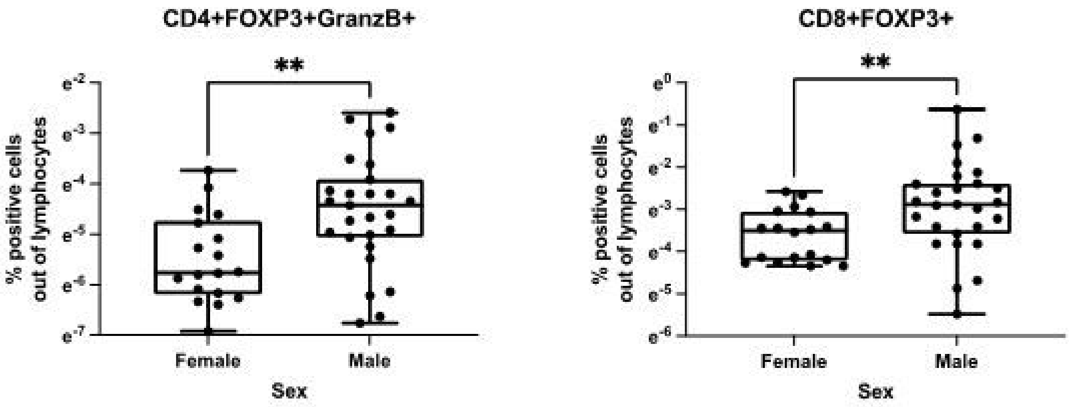

**Figure 4. Effect of sex on Treg distribution in infancy.** Data were analyzed in 123 HEU and 117 HUU. There was a significant effect of sex only in HUU at 28 weeks of life. The graphs show the distribution of the Treg subsets identified in the titles in 18 female and 27 male infants. The asterisks indicate nominal p values<0.01 calculated by Wilcoxon rank-sum test. The FDR adjusted p values were 0.09 for the CD4+FOXp3+GranzB+ comparison and 0.04 for the CD8+FOXP3+ comparison.

Effect of microbiota on the differential frequencies of Treg in HEU and HUU

We tested the hypothesis that differences in Treg subsets between HEU and HUU could be explained by differences in the maternal or infant gut microbiota, which we had previously shown to significantly differ between HEU and HUU[32] (**Figure S4**). To address this hypothesis, we correlated the abundances of maternal gut bacterial genera with infant Treg subsets at birth. In parallel, we correlated infant microbiota with Treg subsets at 6, 28 and 62 weeks of life. Both analyses focused on bacterial taxa and Treg subsets that differed between mothers with and without HIV at delivery and/or HEU and HUU infants. The results revealed multiple significant associations (**Figure 5; Table S4)**. The number of associations decreased over time, due in part to the convergence over time of the gut microbiota of HEU and HUU and the reduction in the number of Treg subsets with differential distributions. The Treg subsets that correlated with the abundance of bacterial taxa in the gut expressed CD25, CTLA4, FOXP3, GranzB, IL-10, IL-35, Lag3, PD-1, TGFb, and/orTNFR2. Multiple bacterial taxa correlated with the Treg distribution (**Figure 5, Table S4**), some of which exhibited significant correlations at multiple time points.

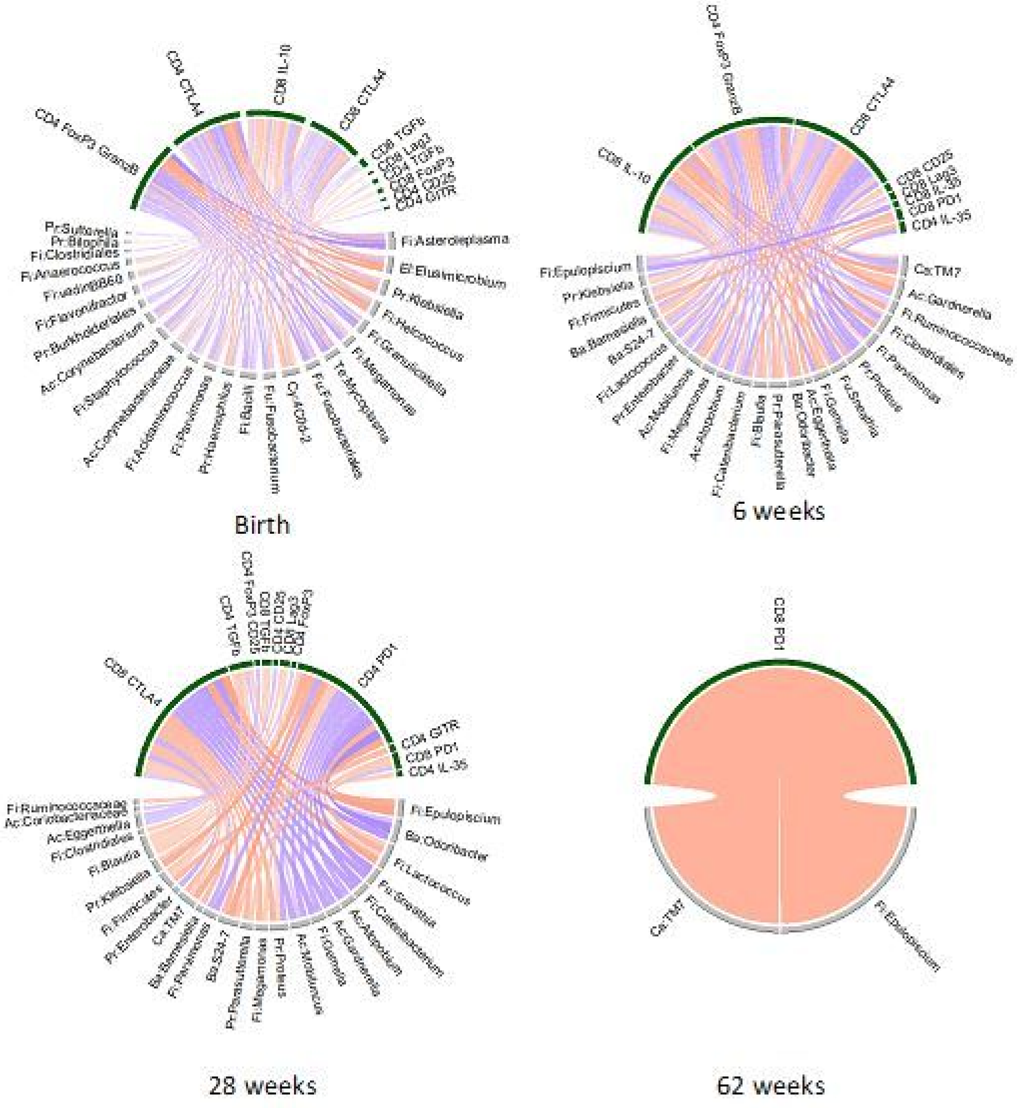

**Figure 5. Chord diagram of associations between Treg subset frequencies and the abundance of gut microbiota that distinguish HEU from HUU.** Data were derived from HEU and HUU with paired Treg and gut microbiome data at the time points indicated on the graph. The Treg subset at birth were correlated with maternal microbiota at delivery. Red chords indicate positive correlations and blue chords negative correlations with FDR $p<0.1$. Treg subsets are clustered on the upper part of the circles (green) and bacteria on the lower part (grey). Rho and p values are listed in **Table S5**.

We further postulated that causal relationships between microbiota and Treg would be reproducible in vitro. To test this hypothesis, we treated PBMC from HUU with bacterial taxa in excess in HEU (Group A), that correlated with specific Treg subsets at multiple time points, and assessed whether any of these Treg subsets were expanded in HUU PBMC. Conversely, we treated PBMC from HEU with bacterial taxa in excess in HUU (Group B). We selected a set of readily available and culturable species including representatives of *Lactococcus*, *Klebisella, Blautia*, and *Ruminococcus* for group A, and *Enterobacter* and *Proteus* for Group B. PBMC from HUU and HEU were treated with UV-inactivated bacterial cultures from Group A and Group B, respectively, at preoptimized conditions consisting of 10 colony-forming units/cell or medium control for 7 days. At the end of the incubation, PBMC were analyzed for select Treg subsets that had shown significant in vivo associations with the bacteria in Groups A or B (Gating strategy in **Figure S5**). PBMC from seven HUU showed significant increases in the proportions of CD4+PD1+ Treg when treated with *L. lactis* or *K. pneumoniae* compared with medium control (**Figure 6**). In addition, CD4+FOXP3+CD25+ Treg significantly increased in *K. pneumoniae*-treated PBMC from HUU**,** CD8+FOXP3+ Treg in *B. wexlerae*-treated PBMC, and CD4+TGFb+ in *R. bromii*-treated PBMC (**Figure 6**). Notably, these in vitro effects largely replicated correlations observed in vivo (**Figure 5** and **Table S4**). In contrast, bacterial treatment did not significantly increase the proportions of any Treg subsets in HEU PBMC with typical examples shown in **Figure S6**.

**Figure 6. Ex vivo treatment of PBMC with bacterial isolates recapitulates in vivo associations with Treg subsets. Left panels:** Data were generated using PBMC from 7 HUU each treated for 7 days with the UV-inactivated bacterial cultures indicated on each graph. P values were calculated with Wilcoxon matched-pairs signed rank test. **Right panels** show typical flow cytometric representations of the data summarized in the left panels. Full gating strategies are shown in **Figure S4**. Please see **Figure S5** for examples of bacterial isolates that did not expand Treg subsets in HEU PBMC.

## Discussion

Compared with HUU, HEU infants exhibit decreased functional immune responses in early life, which may be driven by increased proportions of Treg in HEU. In this study, we identified differences in the distribution of Treg subsets between HEU and HUU in the first year of life and established maternal and infant characteristics associated with the frequencies of Treg subsets. The most prominent factor was the abundance of certain bacterial taxa in the gut microbiome. Gut microbiota have emerged as a central element in the education of local and systemic immune responses [33]. The human gut harbors 12 to 20% of the total lymphocytes and, most importantly, it is a critical site of innate and adaptive T cell maturation, second only to the thymus [34, 35]. Bacterial taxonomic groups, such as segmented filamentous bacteria, *E. coli*, *B. fragilis* and diverse clostridia (e.g. *Ruminococcaceae* and *Lachnospiraceae*) alter the balance between Treg and conventional T cells[36-39]. These studies revealed that bacterial products, such as short-chain fatty acids (SCFA), tryptophan catabolites, and *B. fragilis*-derived polysaccharide A promote Treg differentiation and expansion [40-43]. In addition, experimental in vitro models confirmed that SCFA contribute to Treg differentiation in human PBMC [44, 45]. We previously identified multiple differences in HEU and HUU gut microbiota [32] and demonstrate here that these differences are associated with divergent infant Treg development between these groups.

Notably, differences in the gut microbiota of mothers with and without HIV were associated with differences in Treg frequencies in HEU and HUU cord blood. This finding is in agreement with previous studies showing that the maternal gut microbiome plays an important role in the development of the infant immune system [46-48]. For example, Tanabe et al. showed an association between the maternal gut microbiome and cytokine levels in the cord blood [49] and several studies both in humans and animal models have reported profound effects of the maternal diet on the neonatal immune system mediated by the maternal gut microbiome [46, 50]. The communications between the maternal gut microbiome and the fetal immune system are likely to be assisted by bacterial metabolites that freely cross the placenta[49] and deserve to be further studied.

We postulated that some, but probably not all, associations between gut microbiota and Treg differential frequencies between HEU and HUU reflect direct effects of bacterial taxa on the immune system. For four organisms, higher in HEU than HUU, including select species of *Blautia, Lactococcus, Klebsiella* and *Ruminococcus*, we confirmed their direct relationship with the expansion of Treg using an in vitro model. For two other microorganisms, higher in HUU than HEU, *Proteus* and *Enterobacter*, we could not demonstrate similar relationships.

*Blautia* is a Firmicute belonging to the family of *Lachnospiraceae* [51] and an SCFA producer [51]. *Blautia*'s secondary metabolites and their relationship with the composition of the gut microbiome in human health and disease have raised interest in understanding its physiological properties as well as local gut conditions that modulate its growth, including diet [51, 52]. In our previous study we found that *Blautia* was more abundant in the gut microbiomes of mothers with HIV compared to those without HIV and its abundance positively correlated within mother-infant dyads [32], suggesting that HEU acquired the bacteria directly from their mothers or through shared local conditions in the gut. Recent studies document that people with HIV have a gut microbiome signature characterized by bacterial networks in which *Blautia* represents one of the main nodes [53]. In contrast to people with uncontrolled HIV replication, whose gut microbiotas are enriched in *Blautia*, in elite controllers the gut microbiome is reported to be depleted of *Blautia* [54]. Moreover, the abundance of *Blautia* in the gut microbiome was associated with T cell activation and a variety of neurologic disorders in people with uncontrolled HIV replication [54, 55]. In our study, the abundance of *Blautia* sp. was associated in vivo with increased expression of FOXP3, GranzB, CTLA4, PD1, TGFb and Lag3. In vitro, *B. wexlerae* expanded CD8+FOXP3+ Treg. Collectively, these observations suggest that *Blautia* may play a role in the immunologic dysfunctions observed for HEU. Although not addressed in this work, HEU also have neurologic abnormalities[56] that may be the object of future studies in association with the abundance of *Blautia*. Collectively, these data support conducting future studies of dietary interventions that may modulate the abundance of *Blautia* in the gut microbiome of HEU and/or of pregnant women with HIV.

*Lactococcus* is also a Firmicute (family *Streptococcaceae*). It produces acetate in low glucose conditions, which may contribute to T cell differentiation along the Treg pathway. In fact, *L. lactis* was associated with Treg induction in several animal models [57-59]. Also in animal models, dietary gluten reduced the abundance of *Lactococcus* in the gut [58]. Less is known about the relationship of *Lactococcus* with Treg in humans. In people with HIV, *Lactococcus* has not stood out in the composition of the gut microbiome but was the second most common microbe identified in serum of ARV-treated individuals[60]. In our study, *Lactococcus* was present in higher abundance in HEU than HUU but not in the mothers with HIV compared to those without HIV [32].  In vivo, *Lactococcus* was associated with increased Treg expressing PD1, TGFb, and CTLA4. In vitro, PBMC treatment with *Lactococcus* significantly expanded CD4+PD1+ Treg, partially reproducing the in vivo observation.

*Ruminococcus* is a Firmicute belonging to the family of *Clostridiales*. There are several species of *Ruminococcus* each associated with different clinical disorders. In general, *Ruminococcus spp.* are butyrate producers and, therefore, have the ability to stimulate Treg differentiation [61, 62]. However, high abundance of *R. gnavus* has been associated with inflammatory disorders such as ulcerative colitis and Crohn's disease [63]. There is conflicting information regarding the abundance of *Ruminococcus* in people with HIV [64-67]. In our previous study, we did not find differences in the abundance of *Ruminococcus* in mothers with and without HIV. Nevertheless, *Ruminococcus* had higher abundance in HEU than HUU and correlated in vitro with CD4+ and CD8+ Treg expressing IL10 or IL35. In vitro, *R. bromii* expanded the CD4+TGFb+ Treg, which, like the IL35+ Treg, had significantly higher frequencies in HEU than HUU at 28 weeks of life.

*Klebsiella*, a member of the phylum Proteobacteria, had higher abundance in the gut microbiomes of HEU than HUU and of mothers with HIV than mothers without HIV with significant correlations within mother-infant dyads [32]. *Klebsiella* is uncommon in the gut microbiomes of breastfeeding infants[68]. In contrast, it is regularly found in the gut microbiomes of adults with HIV [69, 70]. Moreover, *Klebsiella* was shown to potentially contribute to the enhanced inflammatory profile of people with HIV and to their neurocognitive impairment [69-71]. In vivo, *Klebsiella* was positively associated with higher frequencies of CD4+FOXP3+GranzB+ as well as CTLA4- and PD1-expressing Treg. In vitro, *K. pneumoniae* also expanded CD4+PD1+ Treg.

Collectively, these observations suggest that high abundance of *Klebsiella* in the HEU gut microbiome may contribute to their immunologic dysfunctions and may warrant studies of interventions to decrease its representation in the gut of HEU and/or their mothers.

Another factor associated with the excess of Treg in HEU was maternal CD4+ cell numbers. This association was present at delivery, suggesting that in utero communications between mother and fetus constituted the underlying mechanism. CD4+ T cell depletion in people with HIV is largely explained by immune activation in addition to the viral cytopathic effect [72]. There is active communication through the placenta between the maternal and fetal immune systems that may explain the effect of maternal immune activation on the fetal immune responses. We have previously shown that mothers with HIV have increased circulating inflammatory markers compared to mothers without HIV at delivery and that HEU also have increased plasma inflammatory markers at birth compared with HUU [73]. It is conceivable that Treg expand in HEU in utero to mitigate inflammation and immune activation derived from the mother. This notion is supported by our previous observation that plasma inflammatory markers positively correlate with the frequencies of Treg subsets in pregnant women with HIV [74].

We found a couple of Treg subsets that were significantly higher in male than female HUU at 28 weeks of life but not in HEU. Higher Treg in males than females is in line with the observation that adult and adolescent males have higher Treg than their female counterparts [75]. Less is known about the relationship of Treg frequencies and sex in prepubertal children. Some evidence suggests that male fetuses have higher proportions of Treg than female fetuses [76]. Male fetuses produce testosterone that acts on T cell receptors [76] and may contribute to the difference in the abundance of Treg between male and female fetuses. Steroid hormones, including testosterone, rapidly decrease in the first 6 months of life and their production resumes only between 6 and 11 years of age [77, 78]. We found differences between male and female HUU at 28 weeks of age but not at birth and not in HEU. Thus, this observation needs additional confirmatory studies.

We did not find that differential DNA methylation of CD4+ T cells played a role in the Treg variance between HEU and HUU. However, this finding does not rule out a contribution of other epigenomic mechanisms (e.g., differences in the DNA accessibility), which deserve to be also investigated.

The study of the inter-relationship between Treg subsets in HEU and HUU was meant to reveal any differences in the coordination of the immune response. However, by and large, the Treg subset correlations were similar in HEU and HUU suggesting that feed-back mechanisms between Treg subsets were preserved in HEU.

Our study had both limitations and strengths. The number of infants with Treg measurements decreased from delivery to 62 weeks of life. Nevertheless, our study has the largest cohort of HEU and HUU and the longest follow up for the comparison of the frequency of Treg in the two groups and for the association of immunologic with gut microbiome differences between groups. A strength of this study was the in vitro verification of microbiome-immune interactions initially identified by in vivo associations.

In conclusion, our study established that the frequencies of Treg subsets differ in HEU and HUU from birth to 62 weeks of life, including an absolute excess of Treg in HEU between 28 and 62 weeks of life. We have previously shown that higher proportions of Treg in HEU compared with HUU were associated with decreased conventional CD4+ T cell function[29], suggesting that similar associations during infancy may underlie the increased susceptibility to infections of HEU. Factors associated with Treg development that may be modified through interventions are the infant and maternal gut microbiomes and maternal inflammation.

## Methods

*Sex as a biological variable.* The distribution of males and females among infants was random and as expected for the birth cohort (**Table 1**). The effect of sex on Treg distribution was analyzed both in HEU and HUU.

*Study design and approval*. The study was approved by the Human Research Ethics Committee at the University of the Witwatersrand (approval number: M171185) and the Colorado Multiple Institutions Review Board (COMIRB 17-0306). Written informed consent was received prior to participation in the study. Women with and without HIV were recruited during labor at Chris Hani Baragwanath Academic Hospital in Johannesburg, South Africa. Inclusion criteria for all women were singleton term gestation, planned vaginal delivery, and intent to breastfeed. Women with HIV had to have been prescribed antiretrovirals but not cotrimoxazole during pregnancy. After signing informed consent, maternal and infant metadata were collected from medical records and by interviewing the study participants at 6, 28, and 62 Weeks postpartum study visits. Maternal blood was obtained at delivery for CD4+ T cells and HIV plasma RNA measurements at the local laboratory. Infant cord blood and peripheral blood obtained at 6, 28 and 62 weeks of life was used to measure Treg subsets. Infant rectal swabs obtained at 6, 28, and 62 weeks of life were used for microbiome analysis.

*Treg characterization by flow cytometry*. Cryopreserved PBMC/cord BMC (CBMC) were thawed, counted, and processed immediately for phenotypic assessment using two staining panels. Panel A consisted of surface staining with Zombie yellow (viability), CD25 FITC (BioLegend), Lag3 PE (Invitrogen), CTLA4 PE-CF594 (BD Biosciences), CD4 PerCP-Cy5.5 (BD Biosciences), CD3 Ax700 (BD Biosciences) followed by fixation and permeabilization using the eBioscience Foxp3 / Transcription Factor Staining Buffer Set (eBioscience). Intracellular staining was then performed with FoxP3 Ax647 (BD Biosciences), Granzyme B APC-fire750 (BioLegend), IL-10 BV421 (BioLegend) and TGFβ PE-Cy7 (BioLegend). Panel B consisted of surface staining with Zombie yellow (viability), CD4 FITC (BioLegend), CD3 PE-CF594 (BD Biosciences), GITR PerCP-Cy5.5 (BioLegend), TNFR2 PE-Cy7 (BioLegend), CD39 Ax700 (R&D Systems), PD1 APC-Cy7 (BioLegend) and TIGIT BV421 (BioLegend). Intracellular staining consisted of FoxP3 Ax647 (BD Biosciences) and IL-35 PE (BioLegend). Analysis was performed using the Galios instrument (Beckman Coulter).

*Ex vivo induction of Treg. Blautia wexlerae* (Cat# BAA-1564, ATCC)*, Lactococcus lactis* (Cat# 19435, ATCC) and *Ruminococcus bromii*(Cat# 27255, ATCC) were subcultured onto Brucella agar plates (Cat# RO1253, Remel) and incubated at 37°C in an anaerobic chamber until colony growth was observed. *Klebsiella pneumoniae, Enterobacter cloacae*, and *Proteus mirabilis* clinical isolates from the Microbiology Clinical Laboratory at the University of Colorado Hospital were subcultured onto sheep blood agar plates (Cat# RO 1202, Remel) and incubated overnight at 37°C. A bacterial suspension for each organism was performed by transferring bacterial colonies to sterile saline and equilibrated to a 0.5 McFarland standard as measured by a turbidity meter [79]. Bacterial suspensions were UV-inactivated for 15 min, aliquoted, and stored frozen at -80°C until use. For stimulation assays, cryopreserved PBMC were thawed, washed, counted with a Guava EasyCyte instrument (Luminex), and resuspended in RPMI 1640 (Corning) containing 10% FBS (Gemini), 2mM L-glutamine (Gemini), 20mM Hepes buffer (Corning), and 1% penicillin/streptomycin solution (Gemini) at $10^6$ PBMC/mL. Cells were incubated with bacteria in pre-optimized conditions at a multiplicity of infection of 10 colony-forming units per viable PBMC for 7 days at 37°C in a $CO_2$ incubator (Thermo-Fisher).During the last 16 h of incubation, Brefeldin-A (5μg/ml, Sigma) was added after which cells were washed with PBS (Corning) and stained with Zombie Aqua Fixability dye (BioLegend), washed with PBS+1%BSA (Millipore Sigma) and stained for surface markers with GITR BV711, PD1 BV785, Lag3 APC-Cy7 (BioLegend), CD4 PerCP-C75.5, CD25 PECF594, CD3 Ax700 (BD Biosciences) in BD Horizon Brilliant Stain Buffer Plus (BD Biosciences). Cells were then washed and fixed/ permeabilized with the eBioscience FOXP3/ Transcription Factor Staining Buffer Set (Invitrogen). Cells were washed with the kit-provided buffer and stained for intracellular markers with IL-10 BV421, CTLA4 BV605, Granzyme B FITC, FOXP3 PE, TGFβ PE-Cy7 (BioLegend), and IL-35 APC (R&D Systems) in BD Horizon Brilliant Stain Buffer Plus. Cells were then washed and resuspended in PBS+1% paraformaldehyde before acquisition on the NovoCyte Quanteon cytometer (Agilent). PBMC from a study-dedicated leukopack control were used in each run to ensure inter-assay reproducibility. Data analysis was performed in FlowJo (BD Biosciences).

*Microbiome profiling.* Fecal microbiota were profiled by 16S rRNA gene sequencing as a component of our previously reported study [32]. All sequences and corresponding metadata were deposited in the NCBI Sequence Read Archive under BioProject accession number PRJNA816484.

*Statistical analysis.*

Treg subset group comparisons: Relative frequencies were used to identify differences in HEU and HUU infants at birth, 6 weeks, 28 weeks, and 62 weeks. These cross-sectional analyses used Wilcoxon rank sum tests from the rstatix package [80] and FDR was used to correct for multiple comparisons for each visit. Additional analyses looking at the effects of covariates like sex and viral load (<50 or >50) were conducted as described above. Spearman's rank correlation coefficient was used to quantify the strengths of the relationships between variables like birthweight and CD4 count and the various Treg subsets. Longitudinal analyses of flow cytometry data used relative frequencies for each cell type and were modeled with a linear mixed effects model (LMM) to account for repeated measurements within each infant. The LMM assumed a normal distribution for the relative frequencies, used a random intercept for infant ID, and included terms for exposure, time (treated as categorical to allow more flexibility) and the interaction between exposure and time. Differences in trends between groups were determined by evaluating F-tests for the exposure by time interaction. An FDR threshold of <0.1 was used to determine statistical significance for all tests. All analyses were conducted using R version 4.1.3 [81].

Correlation Analyses were used to investigate the relationships between the Treg subsets in HEU and HUU infants at birth, 6 weeks, 28 weeks, and 62 weeks. The analysis was conducted in R and Spearman's rank correlation coefficient was used to quantify the strengths of the relationships. Network plots were constructed using igraph [82] and ggraph [83] packages. Spearman correlation analyses were also used to identify relationships between the microbiome and the Treg subsets. All significant microbiome from our previous analysis [32] were used to compare with the Treg data at 6 weeks, 28 weeks, and 60 weeks. The maternal microbiome was also used to correlate with infant Treg data at birth. The Treg data was modeled using HIV exposure status as a confounder and the residuals of the model were used to look at the relationship with the microbiome data. Spearman's rank correlation coefficient was used to identify the relationships and an FDR of <0.1 was used to determine statistical significance. All multiple comparison adjustments were done by visit. Chord diagrams were then constructed for visualization using the Circlize package [84].

Methylation analyses: Intensity Data (IDAT) files containing the methylation data were read into R and analyzed using the procedure described by Maksimovic et al. [85]. Quality control showed the average detection p-values were <0.006 and below the described cutoff. To minimize variation between samples, data normalization was conducted using the *preprocessQuantile* method. Filtering was then used to remove the poor performing probes. Probes were removed if they failed in one or more samples (n = 5,741), were on sex chromosomes (n = 10,185), were in known SNPs (n = 28,298), or were known to be cross-reactive (n = 24,688), resulting in a final list of 796,947 probes. Differential methylation analysis was then used to identify differences in CpG sites between HEU and HUU. M-values were calculated using the lmFit function from the limma package [86] and an FDR cutoff of <0.1 was used to determine significant differences. To interpret the significant CpG sites, gene ontology testing was conducted. In addition to identifying differentially methylated CpG sites, an analysis of differentially methylated regions was also conducted using the DMRcate package [87].

Ex vivo induction of Treg: Nonparametric paired comparisons of bacterial-treated and untreated conditions were performed using Prism 10.1.1 for MacOS software (GraphPad).

## Declarations

**Ethics approval and consent to participate:** This study was approved by the Human Research Ethics Committee at the University of the Witwatersrand (approval number: M171185) and the Colorado Multiple Institutions Review Board (COMIRB 17-0306). Written informed consent was received prior to participation in the study.

**Consent for publication:** Not applicable.

**Availability of data and materials:** 16S rRNA sequences and corresponding metadata were deposited in the NCBI Sequence Read Archive under BioProject accession number PRJNA816484. Data and code for the statistical analysis is available at https://zenodo.org/records/10498652 and https://zenodo.org/records/10498556. Supporting data values for graphs are included in the supplemental materials. Additional data are available by request from AW.

**Competing interests:** The authors declare that they have no competing interests related to this manuscript.

**Funding:** The study was supported by NIAID grant U01AI131360-01 (AW and SAM MPIs) from the National Institute of Allergy and Infectious Diseases.

**Author contributions**: MJJ designed flow cytometry assays, performed statistical analyses; SKL, AEB, and ATS performed immunologic assays; CER, JMK performed microbiome assays; SL performed molecular assays; BMC oversaw bacterial cultures; MCN and SAM oversaw enrollment and follow up of participants; DNF oversaw microbiome assays; AW conceptualized and designed the study, oversaw immunological and molecular assays, participated in the statistical analysis, wrote the manuscript; all authors reviewed and approved the manuscript.

**Acknowledgements:** Not applicable

## Figures

**Figure 1. Comparison of Treg subset frequencies in HEU and HUU.** Data were derived from a longitudinal cohort of 123 HEU and 117 HUU. Treg subsets listed on the y ordinate were compared between HEU and HUU using Wilcoxon rank-sum test. The dots represent differences in each Treg subset at the time points indicated on the graph (top). N (bottom) indicates the number of HEU in red font and HUU in blue font that contributed data at each time point. Red dots indicate significant differences with FDR-adjusted $p<0.1$. The size of each dot is inversely proportional to the unadjusted p value. The distance between each dot and 0 is proportional to the size of the estimated difference. Please see **Figure S2** for the gating strategy and **Table S2** for a listing of medians and p values.

### Figure 1

See image above for figure legend.

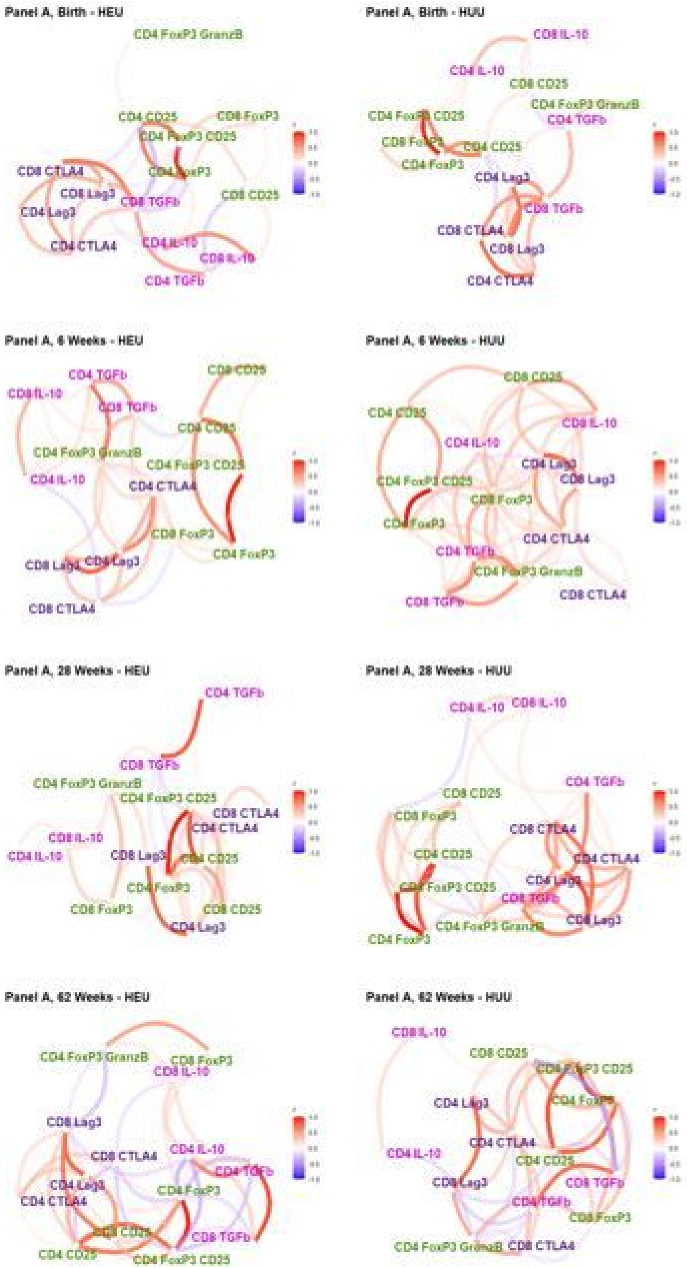

**Figure 2. Relationship between Treg subsets in HEU and HUU.** Data were derived from 29 to 100 participants per group. Red cords indicate positive correlations and blue negative correlations. The color intensity is proportional to the absolute magnitude of the Spearman correlation coefficient. Treg subsets belonging to the same cluster are identified by the color of their names. Most clusters are conserved across time points in HEU and HUU. Please **Figure S3** for correlations of additional Treg subsets.

### Figure 2

See image above for figure legend.

### Figure 3

See image above for figure legend.

### Figure 4

See image above for figure legend.

### Figure 5

See image above for figure legend.

**Figure 6. Ex vivo treatment of PBMC with bacterial isolates recapitulates in vivo associations with Treg subsets. Left panels:** Data were generated using PBMC from 7 HUU each treated for 7 days with the UV-inactivated bacterial cultures indicated on each graph. P values were calculated with Wilcoxon matched-pairs signed rank test. **Right panels** show typical flow cytometric representations of the data summarized in the left panels. Full gating strategies are shown in **Figure S4**. Please see **Figure S5** for examples of bacterial isolates that did not

#### Figure 6

See image above for figure legend.

### Supplementary Files

This is a list of supplementary files associated with this preprint. Click to download.

- HEUTregflowsupplemental11724.docx
